# Estimating treatment effects in trials with outcome data truncated by death: A case study on aligning estimators with estimands

**DOI:** 10.1177/17407745251360645

**Published:** 2025-10-04

**Authors:** Tra My Pham, Brennan C Kahan, Andre Lopes, Memuna Rashid, Peter J Hoskin, Ian R White

**Affiliations:** 1 MRC Clinical Trials Unit at UCL, London, UK; 2 Cancer Research UK & UCL Cancer Trials Centre, London, UK; 3 Mount Vernon Cancer Centre, Northwood, UK; 4 Division of Cancer Sciences, The University of Manchester, Manchester, UK

**Keywords:** Data truncation by death, RCTs, intercurrent events, estimands, estimation, missing data

## Abstract

**Background/Aims:**

Randomised clinical trials assessing treatment effects on health outcomes (e.g. quality of life) can be affected by data truncation by death, where some patients die before their outcome measure is assessed and their data become undefined after death. The ICH E9(R1) addendum on estimands discusses four strategies for handling such terminal intercurrent events: hypothetical, composite, while-alive, and principal stratum. While the addendum emphasises the importance of aligning statistical methods of analysis (i.e. estimators) with estimands, it does not provide specific guidance and consideration on the choice of estimators in practice. We aim to (1) demonstrate how some statistical methods commonly used in trials can be used to estimate different intercurrent event strategies for handling data truncation by death; and (2) describe how missing outcome data (e.g. due to missed assessments or loss to follow-up) can be handled for each estimator.

**Method:**

We use data from SCORAD, a non-inferiority randomised trial comparing single-fraction versus multifraction radiotherapy on ambulatory status at 8 weeks (primary outcome) among patients with spinal canal compression from metastatic cancer. Here, we estimate the effect of radiotherapy on quality of life (secondary outcome), quantified by the difference in mean global health status between the two groups at 8 weeks. We outline the strategies for handling death and describe a selection of commonly used estimators corresponding to each strategy. The handling of missing data is considered and demonstrated as part of the estimation process.

**Results:**

The hypothetical strategy, targeting a treatment effect assuming patients had not died, can be estimated using linear mixed models (a likelihood approach) or multiple imputation (a method commonly used for handling missing data). The composite and while-alive strategies relate to the ‘outcome’ attribute of the estimand; the former incorporates death into the definition of the primary outcome, the latter only uses outcome data before death. These can be estimated by re-defining the outcome, for example, assigning a value reflecting poor global health status post-death, or using the last global health status observed before death. The principal stratum strategy, targeting a treatment effect among patients who would not die under either treatment, can be estimated by an analysis of survivors under specific assumptions. Missing data can be handled with linear mixed models or multiple imputation.

**Conclusions:**

Regarding death as an intercurrent event in the process of defining the estimand for the trial will help clarify the choice of suitable estimators. When choosing the estimators, it is important to consider the assumptions required by the estimators as well as their plausibility given the setting of the trial.

## Background

In randomised clinical trials comparing the effect of different interventions on a health outcome (e.g. quality of life), patients may die before the primary time point at which their outcome is to be assessed, after which point their data no longer exist or are truncated by death.^1–4^ This is problematic because it is not clear how outcomes should be compared between randomised groups.^5^

The ICH E9(R1) addendum on estimands provides a structured framework for defining estimands in trials.^6,7^ Deaths (and other terminal events) are referred to as a type of intercurrent events, i.e. events that occur after randomisation that affect either the interpretation or the existence of outcome associated with the clinical question of interest. The handling of intercurrent events is one of the five attributes that must be specified when constructing an estimand (alongside patient population, treatment conditions, outcome, and population-level summary). Data truncated by death are different from missing data, for example, due to patients not attending all follow-up visits or not completing some questionnaires. The former are undefined after death and must be considered when defining the estimand, while the latter exist but are not observed and need to be addressed in statistical analysis.

The addendum describes four strategies for handling death when defining the estimand: hypothetical, composite, while-alive, and principal stratum; each corresponding to a different treatment effect. While there is an emphasis on aligning statistical methods of analysis (i.e. estimators) with estimands, the addendum does not provide specific considerations for the choice of estimators in practice. This lack of guidance may lead to the adoption of estimators which do not target clear estimands, or which may target estimands other than the one desired.

Prior to the introduction of the addendum, estimands were not stated explicitly in most clinical trial reports, but the estimand could often be inferred from the statistical analysis performed.^8,9^ The handling of data truncation by death has generally been viewed as a statistical analysis task, and as a result it could lead to ambiguity around what treatment effect or estimand was being estimated.^10,11^ Indeed, a recent systematic review of trials published in high-impact general medical journals found that the intercurrent event whose handling was most often not inferable was death.^8^ In many cases, this was because patients who died were often excluded from the analysis, which does not correspond to targeting a clear estimand.^8^ In addition, sometimes data truncated by death are treated as missing data; for example, a trial might be analysed using a method that handles data truncated by death as if they were missing data, resulting in a treatment effect that may not be clinically relevant.^9,12^

The purpose of this article is, therefore, to describe how statistical estimators commonly used in trials can be used to estimate different intercurrent event strategies for handling death, and how these estimators can be adapted to handle missing outcome data. Throughout this article, we assume that we are at the statistical analysis planning stage of the trial, prior to which death has been considered an intercurrent event and the trial team have decided to use one of the strategies in the ICH E9(R1) addendum to handle death when defining the estimand; our aim is to ensure that the analysis targets the estimand strategy used for death. Using data on quality of life in a cancer trial with high mortality as an illustrative example, we outline the different strategies for handling death in defining an estimand. We describe the implementation of some common statistical methods of analysis to estimate each of these strategies and detail their assumptions. We distinguish the issues around data truncation by death from missing data, and handle the latter as part of the analysis process.

## Methods

### Illustrative example: the SCORAD trial

The SCORAD (single-fraction radiotherapy compared to multifraction radiotherapy) trial was a randomised trial assessing whether ambulatory response rate at 8 weeks (primary outcome) among patients with spinal canal compression using single-fraction radiotherapy was non-inferior to multifraction radiotherapy over 5 days.^13^ Briefly, N = 694 patients were randomised (1:1) to the single-fraction or multifraction group. In the multifraction group, N = 8 were found to be ineligible to undergo radiotherapy after randomisation and excluded from analysis. This resulted in a total of N = 686 patients, N = 345 in the single-fraction group, and N = 341 in the multifraction group. The main trial findings have been reported previously.^13,14^

We use, as an illustrative example, data on global health status, a measure of quality of life (secondary outcome) reported at baseline, weeks 1, 4, and 8, using the European Organization for Research and Treatment of Cancer Quality of Life Questionnaire-Core Questionnaire (QLQ-C30) (0 to 100, higher scores reflect better health).^15^ It is a commonly used secondary outcome in cancer trials. We are interested in the difference in mean global health status between the two radiotherapy groups at 8 weeks.

We focus on the handling of death as an intercurrent event. Therefore, for simplicity, we use the same analysis population as that described in the main trial publication and explore different estimand strategies for handling death. Table 1 describes the treatment effects considered in this article, each corresponding to death being handled with the hypothetical, composite, while-alive, and principal stratum strategy, respectively. Briefly, the hypothetical strategy targets the treatment effect in a hypothetical scenario where death had not occurred. The composite strategy incorporates death into the outcome definition, that is, death is considered an unfavourable outcome. The while-alive strategy targets the treatment effect during the period when the patient was alive on the trial. Finally, the principal stratum strategy targets the treatment effect in a group of patients who would survive regardless of which treatment they receive.

**Table 1.** Description of the treatment effects targeted by different strategies for handling death as an intercurrent event in the SCORAD’s analysis of global health status.

Strategy for handling death	What is it?	Full treatment effect description
Hypothetical	The effect of treatment in a hypothetical scenario where death would not occur.	What is the mean difference in global health status at 8 weeks for single-fraction radiotherapy versus multifraction radiotherapy in a hypothetical scenario that patients did not die?^a^
Composite	Death is incorporated in the outcome definition, for example, by assigning a particular value of the outcome to patients who died.	What is the mean difference in global health status at 8 weeks for single-fraction radiotherapy versus multifraction radiotherapy, where patients who died are assigned a global health status score of 0 (the lowest value on the scale) after death?^b^
While-alive	Only values of the outcome prior to death are of interest.	What is the mean difference in global health status at the last point patients were alive, up to 8 weeks, for single-fraction radiotherapy versus multifraction radiotherapy?^c^ What is the mean difference in global health status, averaged over the follow-up period while patients were alive, up to 8 weeks, for single-fraction radiotherapy versus multifraction radiotherapy?^c^
Principal stratum	The treatment effect in the sub-population of patients who would not die under either treatment.	What is the mean difference in global health status at 8 weeks for single-fraction radiotherapy versus multifraction radiotherapy in the sub-population of patients who would survive to 8 ‘weeks regardless of which radiotherapy group they were randomised to?

a This hypothetical scenario may not be well defined for death, particularly in cancer trials.

b This is one possible way that the outcome could be defined using a composite strategy; others include rank-based composite outcomes.^16,17^

c These are two possible ways that the outcome could be defined using a while-alive strategy; others include, for example, weighted average global health status during the follow-up period the patient was alive, with different weightings for time.

We define the visit windows as follows: baseline as days 0 to 6, week 1 as days 7 to 13, week 4 as days 21 to 34, and week 8 as days 49 to 63 from randomisation. For each visit window, we create a categorical variable indicating whether there was (1) at least one record of global health status in the window (i.e. ‘observed’); (2) no record of global health status but the patient was still alive by the end of the visit window (i.e. ‘missing’); or (3) no record of global health status and the patient had died prior to or during that visit window (i.e. ‘truncated’). Our categorisations are simpler than the trial’s definitions, hence results should not be compared directly.^13,14^

Other covariates measured at baseline include age, sex, primary tumour, extent of metastases, number of sites of compression, location of sites of compression, and ambulatory status (Table S1, Supplemental Materials).

### Extent of missing data and data truncated by death

Table 2 summarises the extent of observed, missing, and truncated global health status data by visit window and randomised radiotherapy group. For visit windows containing more than one global health status record, we select the last record for simplicity.

**Table 2.** Number (%) of patients with observed, missing, and truncated global health status data by visit window and randomised radiotherapy group in SCORAD.

	Multifraction (N = 341)			Single-fraction (N = 345)		
	Observed	Missing	Truncated	Observed	Missing	Truncated
Baseline	337 (99%)	4 (1%)	0 (0%)	337 (98%)	8 (2%)	0 (0%)
Week 1	212 (62%)	107 (31%)	22 (6%)	220 (64%)	107 (31%)	18 (5%)
Week 4	164 (48%)	115 (34%)	62 (18%)	160 (46%)	97 (28%)	88 (26%)
Week 8	122 (36%)	90 (26%)	129 (38%)	126 (37%)	81 (23%)	138 (40%)

While truncation by death is always monotone (i.e. after a patient has died their outcome data are truncated for all remaining visit windows of the trial), in SCORAD missing data are non-monotone (e.g. a patient might have a global health status record in weeks 1 and 8 but not week 4).

All other covariates collected at baseline are fully observed, apart from location of sites of compression which is missing for 2 (0.3%) patients.

### How different treatment effects could be estimated with commonly used statistical methods of analysis

We present a selection of statistical methods, or estimators, commonly used in trials to estimate different treatment effects with data truncation by death in SCORAD. We also identify their assumptions and comment on their suitability. Stata code for implementation is included in the Supplemental Materials.

#### Hypothetical strategy

When death is handled by a hypothetical strategy, the estimand of interest is the effect of radiotherapy on global health status had the patients not died. A hypothetical strategy for death may not be well defined as it is unclear how this avoidance of death is to be achieved, particularly in cancer trials.^9^ Nevertheless, here we describe two estimators that could be used to estimate this treatment effect together with their assumptions, should trialists still decide to use this strategy to handle death in their trials.

#### Linear mixed models

Linear mixed models is a natural approach for estimating the hypothetical treatment effect. These models utilise the correlations between repeated measurements of the outcome over time and handle missing data before death and hypothetical data after death in the same way, thus assuming that the patients who had died were still alive. No explicit imputations of unobserved outcome data are created.

Mixed models assume data are missing at random (MAR). Thus, given covariates in the models, outcomes among those who died are assumed to have the same distribution as outcomes among those who did not die. This method is a valid estimator for estimating the hypothetical strategy if all confounders of the association between death and the hypothetical outcome data are included in the model.

A linear mixed model can be fitted to global health status data, conditional on an indicator of visit window for weeks 1, 4, and 8, and an interaction between randomised radiotherapy group and visit window, to allow for both the control mean outcome and treatment effect to vary by visit window. Baseline covariates can be included in the model with or without interactions with visit window. Here, global health status and ambulatory status are included in the model as main effects and interactions with visit window to reflect that baseline values of these outcomes are likely to be more strongly correlated with early outcomes than with later outcomes; other baseline covariates are included as main effects only. The treatment effect at 8 weeks can be extracted directly from the model parameter estimates for the interaction between randomised treatment and visit window. In SCORAD, this model is fitted to N = 539 patients who have at least one record of post-baseline global health status.

#### Multiple imputation

Since we do not observe the hypothetical outcome data after death and these data are required to estimate the hypothetical treatment effect, they could be treated as ‘missing’ and imputed to answer the question ‘what would the patient’s global health status be if they were still alive?’. Multiple imputation is widely used for handling missing data and is therefore a natural approach that could be used to recreate these missing hypothetical data. Because the SCORAD dataset contains both missing data and data truncated by death, the advantage of multiple imputation in this application is that both types of data could be imputed in the same procedure.

Multiple imputation involves defining an imputation model to impute the missing values several times based on information in the observed data, thus creating multiple completed datasets. The planned analysis is then carried out identically in each of the completed datasets, resulting in multiple treatment effect estimates and their associated standard errors. These estimates are then pooled together using Rubin’s rules to obtain a single treatment effect estimate and standard error for inference.^18^

The standard implementation of multiple imputation assumes that missing data are MAR, conditional on the covariates included in the imputation model. To estimate the hypothetical strategy, this means that all confounders of the association between death and the hypothetical outcome data are included as covariates in the imputation procedure (i.e. the ‘no unmeasured confounding’ assumption).^10^

In the SCORAD dataset, both missing global health status data before death and hypothetical global health status data after death can be imputed using multivariate imputation by chained equations, or mice, which is readily available in common statistical software packages.^19^ Here this imputation procedure is implemented in a way that does not distinguish between truncated and missing data. Global health status data in different visit windows (weeks 1, 4, 8) can be treated as separate variables and imputed sequentially, with each visit window being imputed conditional on observed and imputed data from all other visit windows. Several cycles of this procedure are carried out until convergence is reached, at which point one imputation is produced. We use predictive mean matching for the conditional imputation models in order to preserve the observed distribution of global health status,^20^ where the missing/truncated global health status for each patient is imputed with an observed value from five nearest neighbours in each visit window. Imputation is conditional on all baseline covariates (Supplemental Table A1) including baseline global health status. To make the imputation procedure more similar to the linear mixed model described earlier, a randomised radiotherapy group is included as a main effect in the imputation model. An alternative could be to perform multiple imputation stratified by a randomised group to allow for its interactions with covariates in the imputation model. Prior to running mice to impute missing global health status at weeks 1, 4, and 8, missing values in baseline global health status and location of sites of compression are mean-imputed, following a recommendation by White and Thompson.^21^

We perform M = 90 imputations with C = 20 cycles; the number of imputations is chosen to be greater than the percentage of incomplete cases.^20^ After imputation, the treatment effect is estimated among all patients (N = 686) by fitting a linear regression model on global health status at 8 weeks, conditional on randomised group and baseline global health status.

#### Composite strategy

There are different ways to incorporate death into a composite outcome, each leading to a different estimand. In trials like SCORAD where the aim is to assess the effect of treatments on a health outcome (e.g. global health status), one way to form a composite outcome with death (as is often done in cancer trials) is to assign an outcome value that reflects poor health to patients who died.^22^ Another approach is to create an ordered composite outcome where, in the context of the SCORAD trial, the timing of death could be considered first (i.e. earlier death is considered worse than later death), and then among patients who survived to 8 weeks a lower global health status score is considered worse than a higher score.^16^

Here we illustrate the first approach where patients who died are assigned a value of 0, that is, the lowest possible value on the scale, for their global health status after death. In practice, the choice of what this value might be (e.g. 0 or a negative value to reflect quality of life after death to be worse than quality of life when alive) would require clinical evaluation and patient input. We note that assigning different values post-death produces different estimands and therefore results should not be compared as three different estimators of the same estimand.

We handle global health status data that are missing prior to death by multiple imputation. We demonstrate two ways of doing this: (1) the ‘impute then delete’ approach; and (2) the ‘impute conditional on being alive’ approach. Both approaches make the MAR assumption about the missing global health status data. This means that the probability of missingness does not depend on the missing global health status data, conditional on being alive, all observed values of global health status, as well as other covariates included in the imputation model.

#### ‘Impute then delete’

In this approach we impute global health status data at all visit windows both pre- and post-death, then imputed values post-death are deleted and replaced with 0 to form the composite outcome, before the primary analysis is carried out.

The imputation procedure is, therefore, the same as that described earlier for the hypothetical strategy. Once global health status at weeks 1, 4, and 8 has been imputed, the composite outcome is then formed by replacing imputed values for visit windows after death with 0. The analysis model is then fitted to the imputed composite endpoint for all patients (N = 686).

We note that the composite outcome should be formed after multiple imputation, that is, we should not assign scores to visit windows post-death before imputation. This is to avoid situations where a living patient’s global health status is imputed using the assigned score from a neighbour who already died at the visit window being imputed.

#### ‘Impute conditional on being alive’

We can use the same imputation model specification for this approach, but restricting each conditional imputation model such that global health status data at each visit window are only imputed for patients who were alive by that window. The conditional imputation models also need to include as covariates visit-specific indicators of whether the patients had died. Therefore, global health status at week 1 is imputed for participants who were alive by the end of week 1, conditional on their global health status at weeks 4 and 8, as well as indicators of whether they were alive by weeks 4 and 8, respectively.

After imputation, the formation of the composite outcome and primary analysis follow the same procedure described above for the ‘impute then delete’ approach.

#### While-alive strategy

Similar to estimating the composite strategy with missing data, here we handle global health status data that are missing prior to death by multiple imputation. Both multiple imputation approaches described above can be used for this task (also under the MAR assumption). The imputation part for these approaches follows that described previously.

Once we have imputed global health status (both pre- and post-death for ‘impute then delete’, or pre-death for ‘impute conditional on being alive’) at weeks 1, 4, and 8, we redefine the outcome depending on which flavour of the while-alive strategy we are estimating. For the patients’ last post-baseline global health status while alive, we replace the imputed values at visit windows where the patients had died with their observed or imputed global health status at the previous visit window sequentially, since data truncation by death is monotone. For the average post-baseline global health status over the period while the patients were alive, for each patient we could calculate the average of their observed and imputed global health status data for all post-baseline visit windows up to 8 weeks during which they were alive, and use that average as their outcome. The analysis model is then fitted to observed and imputed global health status data up to death in all patients, except those who had died prior to/during the first visit window without having any post-baseline outcome data collected (N = 646).

#### Principal stratum strategy

In two-arm trials such as SCORAD, there are four strata of patients defined based on their potential survival outcomes (Table 3): those who would always survive regardless of randomised treatment (‘always survivors’); those who would survive under one randomised treatment but not the other (the ‘mortality benefiters’ and ‘mortality losers’); and those who would not survive under either randomised treatment (‘always diers’). Global health status at 8 weeks is undefined for those who die. Here we are interested in estimating the treatment effect in the ‘always survivors’ stratum, whose global health status at 8 weeks is well defined under both randomised radiotherapy groups.

**Table 3.** Strata of patients based on potential survival outcomes in SCORAD.

	Survival to 8 weeks		Global health status at 8 weeks	
	Multifraction group	Single-fraction group	Multifraction group	Single-fraction group
Always survivors	Survive	Survive	Defined	Defined
Mortality losers	Survive	Die	Defined	Undefined
Mortality benefiters	Die	Survive	Undefined	Defined
Always diers	Die	Die	Undefined	Undefined

Since we only observe the survival status of patients on their randomised treatment arm (and not their potential survival status had they been randomised to the other arms of the trial), assumptions are required to identify patients who are ‘always survivors’. In SCORAD (and more generally), the stratum of ‘always survivors’ is not the same as the subset of patients who survived to 8 weeks on their randomised treatment during the trial (i.e. the ‘survivors’). The latter is a mixture of ‘always survivors’ and ‘mortality losers’ for the multifraction radiotherapy group, and ‘always survivors’ and ‘mortality benefiters’ in the single-fraction radiotherapy group. Hence, identification of the principal stratum often requires untestable assumptions and estimation can be complex.^23^ However, under the assumption that randomised treatment does not affect mortality (i.e. all patients who die under one treatment would also die under the other treatment, and vice versa), an analysis of survivors can be a valid estimator of the treatment effect among the ‘always survivors’ (our principal stratum of interest).^16^ This assumption is testable, since it implies comparable survival across arms. It seems plausible in SCORAD, since the radiotherapy treatments under comparison did not aim to prolong survival.

As before, we could first handle the missing global health status data prior to death, for example, by either the ‘impute then delete’ or ‘impute conditional on being alive’ approach. Then the primary analysis is performed among patients who survived to 8 weeks (N = 419).

## Results

**Figure 1.**
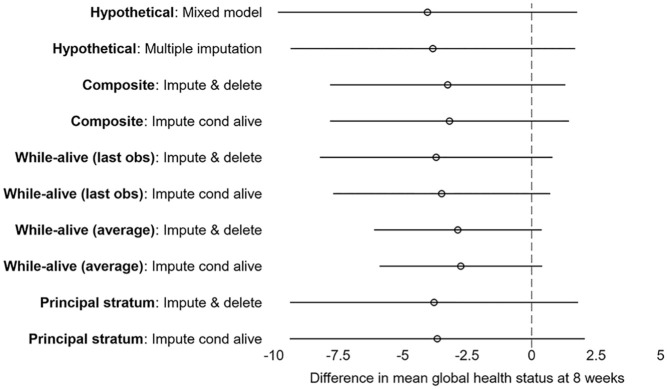
Difference in mean global health status at 8 weeks (single-fraction group minus multifraction group) and 95% confidence intervals for different estimand strategies for handling death and their corresponding estimators in SCORAD.

Figure 1 and Table 4 present the estimated treatment effects for different estimand strategies used for handling death. While the estimated treatment effect varies slightly across the different strategies used for handling death, with the largest effect under the hypothetical strategy and smallest effect under the while-alive strategy where the outcome is the average global health status while alive, inference is generally consistent with no evidence of a difference in global health status between the single-fraction and multifraction radiotherapy groups at 8 weeks. For each estimand strategy, the two flavours of multiple imputation (‘impute then delete’ and ‘impute conditional on being alive’) produce comparable results, except for the first flavour of the while-alive strategy where ‘impute conditional on being alive’ leads to a slightly smaller standard error of the treatment effect compared with ‘impute then delete’. This difference between the two multiple imputation approaches is not observed with different random number seeds. M = 90 imputations provide an appropriate level of precision, with Monte Carlo standard errors of the estimated treatment effect being less than 10% the standard errors.

**Table 4.** Difference in mean global health status at 8 weeks (single-fraction group minus multifraction group) and 95% confidence intervals for different estimand strategies for handling death and estimators in SCORAD.

Intercurrent event strategy		Estimator including handling of missing data	*N*	Estimated treatment effect	Standard error	95% confidenceinterval	
Hypothetical		Mixed model	539	−4.04	2.95	−9.84	1.76
		Multiple imputation(including randomised treatment as main effect)	686	−3.83	2.79	−9.35	1.69
				[0.22]	[0.13]	[0.31]	[0.38]
Composite	Global health status post-death = 0	Multiple imputation:Impute then delete	686	−3.25	2.32	−7.81	1.31
				[0.10]	[0.03]	[0.11]	[0.13]
		Multiple imputation: Imputeconditional on being alive	686	−3.18	2.36	−7.82	1.45
				[0.11]	[0.03]	[0.12]	[0.13]
While-alive	Last observation	Multiple imputation: Impute then delete	646	−3.70	2.29	−8.21	0.81
				[0.14]	[0.05]	[0.16]	[0.19]
		Multiple imputation: Imputeconditional on being alive	646	−3.49	2.14	−7.70	0.72
				[0.11]	[0.04]	[0.13]	[0.14]
	Average	Multiple imputation: Impute then delete	646	−2.86	1.65	−6.11	0.39
				[0.09]	[0.03]	[0.10]	[0.10]
		Multiple imputation: Imputeconditional on being alive	646	−2.75	1.60	−5.90	0.41
				[0.08]	[0.02]	[0.09]	[0.09]
Principal stratum		Analysis of survivors at 8 weeks Multiple imputation: Impute then delete	419	−3.78	2.83	−9.37	1.80
				[0.17]	[0.07]	[0.20]	[0.23]
		Analysis of survivors at 8 weeks Multiple imputation: Impute conditional on being alive	419	−3.66	2.90	−9.38	2.06
				[0.18]	[0.06]	[0.20]	[0.23]

Values in square brackets are Monte Carlo standard errors of multiple imputation results.

## Discussion

### Estimand strategies for handling death and estimators considered

We have identified the strategies for handling death when defining the estimand in randomised clinical trials, and described a selection of statistical methods of analysis that can be used for data truncation by death, when there are also missing data. Using data on global health status, a measure of quality of life in the SCORAD trial, we demonstrated the use of linear mixed models and multiple imputation in estimating the hypothetical strategy. We showed examples of how the composite and while-alive strategies can be estimated by re-defining the outcome. We described the principal stratum of interest, that is, patients who would not die under either treatment, when the principal stratum strategy is used to handle death, and illustrated that treatment effect can be estimated by an analysis of survivors under restricted assumptions.

### Handling of missing data in analysis

We also described how missing data can be handled in the estimation process, either with linear mixed models or multiple imputation. For the estimation of the composite, while-alive, and principal stratum strategies, we illustrated two flavours of multiple imputation, ‘impute then delete’ and ‘impute conditional on being alive’; these have been implemented in a previous cancer trial to impute missing quality of life data with truncation by death.^24^ While these two approaches led to comparable results for each estimand strategy in SCORAD, in our experience ‘impute then delete’ has a slightly more straightforward implementation. However, further work (analytical/simulation studies) is needed to explore whether the imputed values are drawn from similar posterior predictive distributions with these approaches, and whether their statistical properties are comparable.

We focused on estimating strategies for handling death in SCORAD, and thus ignored other intercurrent events for simplicity. However, in practice they may be associated with both missingness and outcome data, for example, early treatment discontinuation may lead to more missing data and worse outcome, and hence need to be taken into account in the analysis to make the MAR assumption more plausible. This is most conveniently done in the multiple imputation approach.

When estimating the hypothetical strategy using SCORAD data, an alternative to the linear mixed model and multiple imputation adjusting for randomised treatment as main effect described earlier is multiple imputation stratified by randomised treatment. These three approaches account for slightly different covariate effects: in particular, multiple imputation stratified by randomised treatment allows for its interactions with baseline covariates included in the imputation model, while the other two approaches do not. The mixed model is therefore less robust to treatment effect heterogeneity than multiple imputation stratified by randomised treatment.^25^

### Other approaches for estimation and considerations

Our choice of estimators presented here was motivated by our aim to demonstrate how practical and commonly used methods can be considered for analysis given their assumptions, in order to align the choice of estimand with estimation. While we did not attempt to present all possible estimators for each estimand strategy, we are aware of other (often more statistically involved) methods that have been discussed in the literature.

#### Hypothetical strategy

Causal inference methods are other candidate estimators for estimating the hypothetical strategy.^26^ In particular, inverse probability of censoring weights could be considered to estimate the hypothetical treatment effect in SCORAD, where the probability of death over time is modelled in order to compute the weights for each patient in each visit window, and the inverse probability weights are then used in the outcome model. However, for this method to work, global health status data that are missing intermittently during follow-up still need to be handled, and if we choose to use multiple imputation for this task then we can proceed to estimate the hypothetical treatment effect using the imputed data without needing to resort to inverse probability weighting.

#### Composite strategy

For the composite strategy, we described an approach for defining the composite outcome where global health status after death could be replaced with a value representing poor quality of life, with different values corresponding to different estimands. An alternative is to use rank-based approaches, such as that described in Colantuoni et al.,^16^ or win ratios.^17^ A common disadvantage of composite outcomes is that the quantification and interpretation of the treatment effect is often less straightforward.

#### While-alive strategy

For the while-alive strategy, an alternative to using the last value of global health status before death could be to obtain a summary of all global health status data prior to death, such as the area under the curve.^27,28^ Here we demonstrated the handling of missing values prior to death using multiple imputation, a principled method that is preferred to *ad hoc* approaches such as ‘last observation carried forward’, or replacing all missing global health status with the worst/best value.

#### Principal stratum

We described the analysis of survivors as an estimator for the treatment effect in the principal stratum of patients who would survive under either treatment, and discussed the assumption required by this estimator for valid inference, which can be restrictive in many settings. The treatments under comparison in SCORAD did not aim to prolong survival; the main trial publication reported that there was no statistically significant difference in survival between treatment groups.^13^ Had the treatments had an effect on survival, such that, for example, survival was prolonged in frail patients, then the analysis of survivors would be a biased estimator of the principal stratum of ‘always survivors’.^16^ Methods discussed in the causal inference literature for estimating the survivor average causal effect, such as the re-weighting approach proposed by Hayden et al.,^29^ could be used to estimate the principal stratum treatment effect. These methods rely on different (and also untestable) assumptions, and therefore should be accompanied by sensitivity analyses.

### Implications and future work

Regarding death as an intercurrent event when defining the trial’s estimand will help clarify the choice of appropriate estimators. Since different estimators can target different estimands, it is important to consider estimators that target the chosen estimand and assess which of these available estimators have the most realistic assumptions given the trial’s setting.

In this article, we aimed to demonstrate how each estimand strategy could be estimated using common analysis methods, once the estimand for the trial has been defined. Since the introduction of the ICH E9(R1) addendum on estimands, there has been an emerging literature around the choice of estimand strategies for handling different types of intercurrent events, including death, in various clinical contexts.^30–40^ This choice should be clinically relevant and driven by the aim of the trial. This is an important consideration that requires input from various stakeholders.^31,41,42^

The scope of this article was on estimating different estimand strategies for death. In practice, in a given trial there will likely be multiple intercurrent events, and the handling of each intercurrent event might be different depending on the aim of the trial and what is clinically meaningful. For further discussions on this topic, readers may refer to some recent work focusing on the estimation of estimands that consist of multiple estimand strategies for different intercurrent events.^43,44^

## Supplemental Material

sj-docx-1-ctj-10.1177_17407745251360645 – Supplemental material for Estimating treatment effects in trials with outcome data truncated by death: A case study on aligning estimators with estimands Supplemental material, sj-docx-1-ctj-10.1177_17407745251360645 for Estimating treatment effects in trials with outcome data truncated by death: A case study on aligning estimators with estimands by Tra My Pham, Brennan C Kahan, Andre Lopes, Memuna Rashid, Peter J Hoskin and Ian R White in Clinical Trials
