# Supplementary material for: Estimating treatment effects in trials with outcome data truncated by death: A case study on aligning estimators with estimands
Source: Clin Trials. 2025 Oct 4;22(6):676–86. doi: 10.1177/17407745251360645 (PMC12647389; doi:10.1177/17407745251360645)
Supplement: sj-docx-1-ctj-10.1177_17407745251360645 – Supplemental material for Estimating treatment effects in trials with outcome data truncated by death: A case study on aligning estimators with estimands [file sj-docx-1-ctj-10.1177_17407745251360645.docx]

# **Supplemental materials**

# Estimating treatment effects in trials with outcome data truncated by death: a case study on aligning estimators with estimands

## Stata code for the SCORAD’s analysis of global health status

Table S1. Variables used in the SCORAD’s analysis of global health status (wide-format data).

| Variable |  | Stata variable name | Values |
| --- | --- | --- | --- |
| Global health status^a^ | Baseline Week 1 Week 4  Week 8 | ghs0 or ghs_bl ghs1  ghs2  ghs3 | 0–100 |
| Data availability indicator for global health status |  | datastatus0  datastatus1  datastatus2  datastatus3 | 1 (observed), 2 (missing), 3 (truncated) |
| Randomised treatment |  | trt | 1 (multifraction), 2 (single-fraction) |
| Other baseline covariates | Age (in years) | age | Continuous |
|  | Sex | sex | 1 (male), 2 (female) |
|  | Primary tumour | primtumour | 1 (prostate), 2 (lung), 3 (breast), 4 (GI), 5 (Other) |
|  | Extent of metastases | extentmet | 1 (nonskeletal metastases absent), 2 (nonskeletal metastases present) |
|  | Number of sites of compression | noscc | 1 (single), 2 (multiple) |
|  | Location of sites of compression | locatscc | 1 (C1–T12), 2 (L1–S2), 3 (T6 –L5) |
|  | Ambulatory status | ambul_bl | Grades 1–4 |

^a^When data are used in long format, global health status is stored in variable ghs with a visit window indicator visit (for weeks 1, 4, and 8); baseline global health status is stored in variable ghs_bl.

### Hypothetical strategy

#### Linear mixed models

Use data in long format, fit a random intercept and slope model with unstructured variance–covariance matrix for global health status.

mixed ghs i.visit 2.trt#i.visit c.ghs_bl##i.visit c.age i.sex i.primtumour i.extentmet i.noscc c.locatscc i.ambul_bl##i.visit || id: visit, stddev cov(uns) iter(20) reml dfmethod(kroger)

Note that this model allows the effects of baseline global health and ambulatory status to vary by visit window, since their correlations with global health status recorded during follow-up are likely to decrease over time.

Display treatment effect at 8 weeks.

display _b[2.trt#3.visit]

#### Multiple imputation

Use data in wide format, perform multiple imputation with M=90 imputations and C=20 cycles.
mi set wide
mi register imputed ghs1 ghs2 ghs3
mi register regular trt ghs0 age sex primtumour extentmet noscc locatscc ambul_bl

mi impute chained (pmm, knn(5)) ghs1 ghs2 ghs3 = i.trt c.ghs0 c.age i.sex i.primtumour i.extentmet i.noscc c.locatscc i.ambul_bl, add(90) burnin(20) rseed(982)

Fit the substantive analysis model in each imputed dataset and combine results using Rubin’s rules.
mi estimate, mcerror: regress ghs3 i.trt c.ghs0

### Composite strategy

#### ‘Impute then delete’

Use data in wide format, perform multiple imputation with M=90 imputations and C=20 cycles.
mi set wide
mi register imputed ghs1 ghs2 ghs3
mi register regular trt ghs0 age sex primtumour extentmet noscc locatscc ambul_bl

mi impute chained (pmm, knn(5)) ghs1 ghs2 ghs3 = i.trt c.ghs0 c.age i.sex i.primtumour i.extentmet i.noscc c.locatscc i.ambul_bl, add(90) burnin(20) rseed(982)

Replace imputed global health status after death with 0 to form the composite ghs3_c0.
forvalues = 1/3 {
 mi passive: generate ghs`v'_c0 = ghs`v'
 mi passive: replace ghs`v'_c0 = 0 if datastatus`v' == 3

}

Fit the substantive analysis model in each imputed dataset and combine the results using Rubin’s rules.
mi estimate, mcerror: regress ghs3_c0 i.trt c.ghs0

#### ‘Impute conditional on being alive’

Use data in wide format to perform multiple imputation. For this procedure Stata requires global health status data at visit windows after death to be replaced with a constant (e.g. -9) to distinguish these from missing data prior to death. The value of the constant is not important and should lead to exactly the same results.
forvalues v=1/3 {
 replace ghs`v' = -9 if datastatus`v' == 3
}

Next define visit-specific death indicators: for each visit window, the indicator takes value 0 if the patient was alive by the end of the visit window, or 1 if the patient died before or during the visit window.
forvalues v=1/3 {
 mark d`v' if datastatus`v' == 3

}

Perform multiple imputation, including these death indicators in the imputation models.
mi set wide
mi register imputed ghs1 ghs2 ghs3
mi register regular trt ghs0 age sex primtumour extentmet noscc locatscc ambul_bl d1 d2 d3

mi impute chained (pmm if !d1, knn(5)) ghs1 (pmm if !d2, knn(5)) ghs2 (pmm if !d3, knn(5)) ghs3 = i.trt c.ghs0 c.age i.sex i.primtumour i.extentmet i.noscc c.locatscc i.ambul_bl i.d1 i.d2 i.d3, add(90) burnin(20) rseed(982)

Replace imputed global health status after death with 0 to form the composite ghs3_c0.
forvalues v = 1/3 {
 mi passive: gen ghs`v'_c0 = ghs`v'
 mi passive: replace ghs`v'_c0 = 0 if datastatus`v' == 3
}

Fit the substantive analysis model in each imputed dataset and combine the results using Rubin’s rules.
mi estimate, mcerror: reg ghs3_c0 i.trt c.ghs0

### While-alive strategy

#### Last observation while alive

After multiple imputation (either ‘impute then delete’ or ‘impute conditional on being alive’, replace global health status after death with the last value before death.

mi passive: generate ghs1_wa = ghs1
mi passive: replace ghs1_wa = . if datastatus1 == 3

mi passive: generate ghs2_wa = ghs2
mi passive: replace ghs2_wa = ghs1_wa if datastatus2 == 3

mi passive: generate ghs3_wa = ghs3
mi passive: replace ghs3_wa = ghs2_wa if datastatus3 == 3

Fit the substantive analysis model in each imputed dataset and combine the results using Rubin’s rules.

mi estimate, mcerror: regress ghs3_wa i.trt c.ghs0

#### Average while alive

Define visit-specific indicators of being alive; for each visit window, the indicator takes value 0 if the patient died before or during the visit window or 1 if the patient was alive by the end of the visit window.
forvalues v=1/3 {
 mark alive`v' if datastatus`v' != 3
}

Calculate the average of the observed and imputed global health status data during the follow-up period up to 8 weeks while the patient was alive.
forvalues v=1/3 {
 mi passive: generate ghs`v'_wa = ghs`v'*alive`v'
}

mi passive: generate avg_ghs_wa = (ghs1_wa + ghs2_wa + ghs3_wa)/(alive1 + alive2 + alive3)

Fit the substantive analysis model in each imputed dataset and combine the results using Rubin’s rules.
mi estimate, mcerror: regress avg_ghs_wa i.trt c.ghs0

### Principal stratum strategy

After multiple imputation, fit the substantive analysis model restricted to patients who were alive by week 8 in each imputed dataset and combine the results using Rubin’s rules.

mi estimate, mcerror: regress ghs3 i.trt c.ghs0 if datastatus3 != 3
